# Perpetrator pose reinstatement during a lineup test increases discrimination accuracy

**DOI:** 10.1038/s41598-021-92509-0

**Published:** 2021-07-09

**Authors:** Melissa F. Colloff, Travis M. Seale-Carlisle, Nilda Karoğlu, James C. Rockey, Harriet M. J. Smith, Lisa Smith, John Maltby, Sergii Yaremenko, Heather D. Flowe

**Affiliations:** 1 School of Psychology, University of Birmingham, Edgbaston, B15 2TT UK; 2 School of Law, Wilson Center for Science and Justice, Duke University, Durham, USA; 3 School of Psychology, University of Kent, Canterbury, UK; 4 Department of Economics, University of Birmingham, Edgbaston, UK; 5 Department of Psychology, Nottingham Trent University, Nottingham, UK; 6 School of Criminology, University of Leicester, Leicester, UK; 7 School of Psychology, University of Leicester, Leicester, UK; 8 Department of Clinical Psychological Science, Maastricht University, Maastricht, The Netherlands

**Keywords:** Psychology, Human behaviour, Learning and memory

## Abstract

We examined how encoding view influences the information that is stored in and retrieved from memory during an eyewitness identification task. Participants watched a mock crime and we varied the angle from which they viewed the perpetrator. In Experiment 1, participants (*N* = 2904) were tested with a static photo lineup; the viewing angle of the lineup members was the same or different from the perpetrator at encoding. In Experiment 2, participants (*N* = 1430) were tested with a novel interactive lineup in which they could rotate the lineup faces into any angle. In both experiments, discrimination accuracy was greater when the viewing angle at encoding and test matched. Participants reinstated the angle of the interactive faces to match their encoding angle. Our results highlight the importance of encoding specificity for eyewitness identification, and show that people actively seek out information in the testing environment that matches the study environment to aid memory retrieval.

Worldwide, witnesses are given lineups to help the police identify criminal perpetrators. A lineup contains the police suspect—who may or may not be the perpetrator—embedded among ‘fillers’, who are individuals who look similar to the police suspect and are known by the police to be innocent. The goal of the eyewitness is to identify the perpetrator if the perpetrator is present in the lineup (known as a correct identification) or to identify no one if the perpetrator is absent from the lineup (known as a correct rejection). The ability of the eyewitness to distinguish the presence or absence of the perpetrator is known as discrimination accuracy. In many countries (e.g., the US, Germany, Canada, Australia), lineups consist of static photographs^1, 2^. Lineup members are shown from the shoulders up, facing forward, even if a witness viewed the perpetrator from a different angle at the time of the crime (e.g., saw only their profile view). The National Academy of Sciences recently called for the development of new technology to improve lineup identification accuracy^3^. In this paper, we heed this call and examine whether discrimination accuracy can be improved by enabling witnesses to see the lineup faces from the same angle that the perpetrator’s face was seen during the crime. We also introduce a novel interactive lineup procedure to test whether during the lineup people spontaneously reinstate the angle at which they saw the perpetrator, and if so, whether pose-reinstatement is associated with increased discrimination accuracy.

There are good reasons to predict that discrimination accuracy will be higher if witnesses can view the lineup faces from the same angle that they studied the perpetrator. One of the most influential principles of human memory—*encoding specificity*—holds that the correspondence in the context in which memories are acquired and retrieved is a powerful determinant of memory accuracy^4^. One example is that divers, who learnt words either on dry land or in water, were better able to recall words if they were tested in the same environment as they had studied the words compared to the alternative environment^5^. The notion that the match between encoding and retrieval is important is also central to other key concepts in memory theory, such as the transfer-appropriate processing framework^6^, and the proceduralist approach, which assumes that encoding operations are re-enacted during remembering^7^. These principles predict that greater overlap of cues present at encoding and test, such as a high correspondence between viewing angle at encoding and test, lead to better memory performance.

In accordance with these memory principles, a wealth of face recognition research shows that similarity across study-test viewing angle improves recognition accuracy^8–10^. At a 30-degree study-test difference, the performance cost plateaus, and at 45 degrees, recognition performance is impaired significantly^11^. Neurophysiological studies also indicate pose-sensitivity in cortical regions^12^. In the context of police lineups, overlapping cues at learning and test, such as the quantity of facial information available at encoding versus test (i.e., internal portion of faces versus full faces)^13^, clothing^14^, and disguises^15^ boost discrimination accuracy. Context reinstatement^16^ and alcohol state-dependent learning^17^ effects have also been reported in the eyewitness literature. Together, this research supports the encoding specificity principle, whereby overlapping cues at learning and test facilitate accurate memory retrieval.

What is not yet clear, however, is whether the encoding specificity principle generalises to conditions in which people encode viewpoint information and use it during a lineup identification test. Face recognition paradigms employ numerous study-test trials. Each study trial presents an individual face, with pose typically varying across faces. In these studies, pose is therefore a key distinguishing feature, so participants may attend to and encode pose information more than they otherwise would in a more naturalistic context^18^. Indeed, some research suggests that in more naturalistic contexts, the correspondence between study and test pose does not always enhance recognition accuracy, and faces at certain angles (i.e., the profile view) are difficult to learn and recognize^10, 19^. Null findings regarding the overlap of pose at learning and test have also been reported in the eyewitness literature. A study that varied whether a perpetrator was studied at eye level versus from overhead found that recognition accuracy was not increased by having matching information at the lineup test. However, this study was likely underpowered^20^. Therefore, it is important to test if people benefit from consistent viewpoint information at study and at test, and under conditions that are akin to real life, such as an eyewitness identification task.

Moreover, research has not yet determined if, during memory retrieval, humans *actively* seek out information at test that matches the study environment as an aide-mémoire. Most memory paradigms experimentally manipulate the degree of overlap between the cues present at encoding and at test, with some participants allocated to experience overlap and others not^5^. But would, for example, divers who studied underwater be more inclined to jump back into the pool at test to reinstate study context to aid their memory retrieval compared to those who studied on land? Here, we test if participants naturally seek out cues at test that correspond with information learned at study, using an eyewitness identification task.

To do so, we developed an interactive lineup procedure, wherein each lineup face can be rotated along the vertical axis. This enables the witness to dynamically view the lineup faces from − 90° to 90° and hold the faces in any pose desired (see https://tinyurl.com/t4nc9gp). If participants reinstate pose at test—by naturally rotating the lineup faces into the same angle from which they saw the perpetrator commit the crime, then this suggests people encode viewpoint information, and seek (consciously or unconsciously) to make use of overlapping cues gleaned through pose reinstatement. Further, if accurate participants reinstate perpetrator pose to a greater degree than inaccurate participants, this suggests pose information is a valuable retrieval cue for eyewitnesses.

To summarise, we ask (1) whether consistent viewpoint information at study and at test is associated with higher discrimination accuracy; and (2) whether people naturally reinstate at test the pose in which they had viewed a perpetrator if given the opportunity to do so with an interactive lineup.

## Experiment 1

In Experiment 1, we tested whether consistent viewpoint information at study and at test improved accuracy on a lineup identification task. Based on the encoding specificity principle and the existing face recognition literature, we predicted that discrimination accuracy would be higher when participants had available at test the same pose information that they encoded compared to when they had different pose information (pose-reinstatement hypothesis). We were also interested whether discrimination accuracy would be higher when participants had available at test the same pose information that they encoded as opposed to different pose information, *and* the highest when they had the same pose information *plus* an additional unstudied pose. Specifically, a geometric representation of the face can be constructed from the frontal and profile views of the face^21^. Indeed, the first police system to catalogue faces for criminal identification was developed by Alphonse Bertillon, and it photographically described arrestees using the profile and frontal view. Seeing a face from more than one angle is thought to be useful for building a representation of the face’s three-dimensional structure, and knowing the three-dimensional structure of a face can provide additional cues that boost discrimination accuracy^9, 22^. In Experiment 1, we tested whether seeing the lineup faces from the angle in which the perpetrator was studied improved discrimination (pose reinstatement hypothesis) compared to when such information was not available, and (b) whether having the same pose information plus additional unstudied information about the face at test boosted discrimination accuracy the most. We pre-registered our hypotheses and analysis plan before we collected data (https://osf.io/vs48c).

## Methods

All methods were carried out in accordance with relevant guidelines and regulations.

### Design

We used a 2 (perpetrator encoding pose: front, right-profile) × 3 (lineup member pose: front, right-profile, front and right-profile) × 2 (target: present, absent) between-subjects design.

### Participants

Participants (*N* = 3021) were recruited using Amazon Mechanical Turk; they were remunerated 0.40 cents to take part in the experiment, which took 5 min to complete. Participants who incorrectly answered the validation question or experienced a technical issue (*n* = 117) were excluded, leaving a total of 2,904 participants (42% female; 18–76 years old, *M* = 37.46, *SD* = 11.95 years; 72% Caucasian, 10% Black or African American, 6% Hispanic, 5% East Asian, 2% South Asian, 2% Other, and 3% prefer not to say). Our data-collection stopping rule was to recruit 3,000 participants—250 in each of the between-subjects conditions. Using the mean difference and *SD*s observed in Mickes, Flowe, and Wixted, 2012^23^ as a guide, a power analysis indicated that, with 250 subjects per between-subjects condition, power would exceed 80%. The research was reviewed according to the University of Birmingham Science, Technology, Engineering and Mathematics Ethical Review Committee. Informed consent was obtained from all subjects.

### Materials

We filmed a mock crime of a Caucasian male perpetrator aged in his 30 s stealing a handbag from behind a female victim. We filmed three versions of the crime: the perpetrator was shown from the front, left-profile, or the right-profile. The whole crime was 14 s in length and the perpetrator’s face was in view for approximately 8 s. For Experiment 1, we used only the front and right-profile videos.

**Figure 1:**
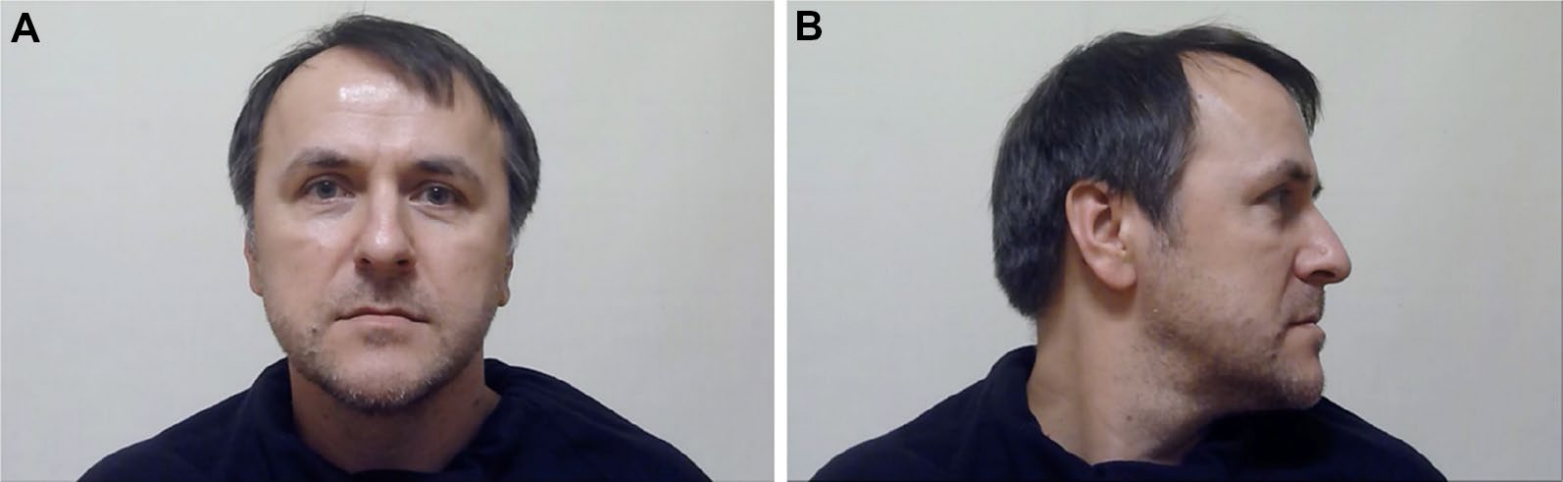
Example of lineup faces from the (**A**) front and (**B**) right-profile. Consent to publish identifying images was obtained.

We recruited 9 members of the public to be the lineup fillers. Following recommended practice, the fillers were individuals who matched the physical appearance (i.e., age, build, gender, skin tone, hair color, eye color, facial hair, hairstyle) of the perpetrator from the video^24, 25^. We took static photographs of each lineup member from the shoulders up showing him facing directly towards the camera in frontal view, and showing him turning away from the camera in profile view (see Fig. 1). Target-absent lineups contained all 9 fillers. Target-absent lineups are akin to the real-life scenario in which the police have apprehended a suspect who matches the description of the perpetrator, but is innocent. Target-present lineups contained the perpetrator and 8 fillers.

To check that our lineup fillers were plausible alternatives to the suspect, we conducted a mock-witness test. First, we asked 10 independent observers to describe the appearance of the perpetrator while looking at the perpetrator’s photograph to create a modal description. We presented a different group of participants acting as mock-witnesses (*N* = 80) with the description of the perpetrator followed by a frontal target-present lineup or target-absent lineup. The mock-witnesses did not view a crime or a to-be-remembered face; they simply had to select the lineup member who best fit the modal description that we provided for them^25^. The perpetrator was identified 7% of the time in the target-present lineup, a rate that did not exceed chance (11%, *p* > 0.05) and the most frequently chosen lineup member was identified 20% of the time in the target-absent lineup, a rate that did not significantly exceed chance expectation. We also calculated E’^26^, a measure of *effective size*, which assesses the number of lineup members that are effective at drawing mock-witness choices^27^. Effective size was 6.50 (95% CI [4.92, 9.61]) in the target-present lineup and 6.76 (95% CI [5.57, 8.60]) in the target-absent lineup. These values are appreciably larger than the effective size of lineups reported in field research on UK police lineups (e.g., Valentine & Heaton, 1999, reported a mean effective size of 4.24 (*SE* = 0.31) for photo lineups, and 4.46 (*SE* = 0.32) for video lineups)^28^. Together, the mock-witness test illustrated that our lineups were perceptually fair, based on the description of the perpetrator.

### Procedure

At the start of the experiment, participants were asked a number of demographic questions (age, sex, ethnicity/race). Then participants watched either the front or right-profile mock-crime video. We told participants to pay attention because they would be asked questions about the video later. Next, participants watched a distractor cartoon for 1 min 11 s and attempted to solve anagrams for a further 2 min. We asked participants if they had experienced any technical problems when viewing the video. Following this, participants were told that they would view a lineup and their task was to try and recognize the perpetrator from the mock-crime video. In line with recommended police practice, participants were told that the perpetrator may or may not be present in the lineup^24^.

Next, the lineup was displayed. The lineup was administered sequentially (i.e., one lineup member was shown at a time). We experimentally manipulated the angle from which the lineup members were shown (see Fig. 1): In the front condition, the lineup members were shown exclusively from the front, whereas in the right-profile condition, they were shown exclusive in right-profile. In the front and right-profile condition, the lineup members were shown from the front as well as in the right-profile, and for each lineup member, the front and right-profile images were shown simultaneously. The order in which the lineup members were presented was randomly determined for each participant. Each lineup member was accompanied by a number corresponding to their position in the lineup (1–9). We asked participants to write down the number of the lineup member they believed to be the perpetrator, if they believed the perpetrator was present in the lineup. Participants saw each face only once and could not review previously seen faces after they had advanced to the next lineup member. Participants could view each lineup member for any length of time desired and once they were finished viewing each member, they pressed a “next” button. After viewing the nine lineup members, participants made an identification decision by selecting a number (1–9), or indicating that the perpetrator was “Not Present.” All participants were asked to rate their confidence in their response on an 11-point Likert-type scale ranging from 0% (not at all sure) to 100% (completely certain). Finally, at the end of the experiment, participants were asked if they had experienced any technical problems while viewing the lineup images, and to select from a drop-down menu the type of crime shown in the video as a manipulation check.

### Conference presentation

Sections of these data were presented by Heather D. Flowe at the Society for Applied Research in Memory and Cognition (June, 2019), Cape Cod, Massachusetts, The United States.

## Results

Our data are available (https://osf.io/jm2k9/). Recall that participants were randomly assigned to encode the perpetrator from the front (*n* = 1449) or the right-profile (*n* = 1455). After watching the mock crime video, participants were randomly assigned to a lineup condition, with 969 viewing the front lineup (480 viewed a target-present lineup and 489 viewed a target-absent lineup), 975 the right-profile lineup (487 viewed a target-present lineup and 488 viewed a target-absent lineup), and 960 the front + right-profile lineup (493 viewed a target-present lineup and 467 viewed a target-absent lineup). For analysis, we combined over perpetrator encoding pose and lineup member pose conditions to create same-pose, different-pose, and same + additional pose conditions, as per our OSF pre-registration. This allowed us to test the pose-reinstatement hypothesis, and whether having the same pose information plus additional unstudied information about the face boosted discrimination accuracy the most. The numbers of participants in each pose condition by experimental group are given in Table 1.

**Table 1:** Frequencies of participants in each pose condition by encoding group and lineup type in Experiment 1.

Lineup type	Same pose (*n* = 969)		Different pose (*n* = 975)		Same + additional pose (*n* = 960)	
	Front encoding	Right-profile encoding	Front encoding	Right-profile encoding	Front encoding	Right-profile encoding
Front lineup	482	–	–	487	–	–
Right-profile lineup	–	487	488	–	–	–
Front + right-profile lineup	–	–	–	–	479	481

Response frequencies for perpetrator, filler and reject (i.e., “Not Present”) decisions at every level of confidence for the same-pose, different-pose, and same + additional pose conditions are displayed in Table 2. The overall correct ID rate of the perpetrator (displayed in the proportion row in Table 2) is equal to the total number of perpetrator IDs from target-present lineups divided by the number of target-present lineups, in each condition. Because there was not a designated innocent suspect, the number of innocent suspect IDs in target-absent lineups was estimated using the total number of filler IDs from target-absent lineups. Following standard practice^29^, this estimate was obtained by dividing the number of target-absent filler IDs by the number of lineup members (i.e., 9). That estimated value was then divided by the number of target-absent lineups to estimate the false ID rate in each condition. The overall correct ID rates were 0.66, 0.49, and 0.65 for the same-pose, different-pose, and same + additional pose conditions, respectively. The corresponding overall false ID rates were 0.04, 0.05, and 0.04 for the same-pose, different-pose, and same + additional pose conditions, respectively. Thus, even without performing any additional analyses, it is clear that those in the same and same + additional pose conditions performed better than those in the different pose condition, as predicted by the pose-reinstatement hypothesis.

**Table 2:** Frequencies of perpetrator, filler and reject identification decisions by pose condition in Experiment 1.

Confidence rating	Same pose					Different pose					Same + additional pose				
	Target-present (*n* = 480)			Target-absent (*n* = 489)		Target-present (*n* = 487)			Target-absent (*n* = 488)		Target-present (*n* = 493)			Target-absent (*n* = 467)	
	Perp	Filler	Reject	Filler	Reject	Perp	Filler	Reject	Filler	Reject	Perp	Filler	Reject	Filler	Reject
0	0	0	0	1	0	0	0	2	1	4	0	0	1	1	2
10	2	0	1	3	3	3	4	0	4	3	1	2	0	4	2
20	3	2	4	7	2	2	2	2	13	2	2	2	2	9	4
30	4	3	3	6	6	3	5	5	9	5	4	1	3	8	3
40	6	3	2	15	9	9	11	6	13	12	3	4	5	14	5
50	16	9	5	24	17	21	13	22	28	19	18	13	8	21	11
60	23	15	5	24	18	27	12	18	35	27	11	8	15	20	21
70	20	11	13	29	33	33	11	29	32	31	33	18	12	27	43
80	50	17	21	38	50	50	10	33	36	64	55	8	21	26	44
90	81	9	21	21	72	46	13	31	19	64	90	11	13	17	80
100	113	8	10	19	92	43	6	15	15	52	103	8	18	16	89
Total	318	77	85	187	302	237	87	163	205	283	320	75	98	163	304
Proportion	0.66	0.16	0.18	0.38	0.62	0.49	0.18	0.33	0.42	0.58	0.65	0.15	0.20	0.35	0.65

### ROC analysis

**Figure 2:**
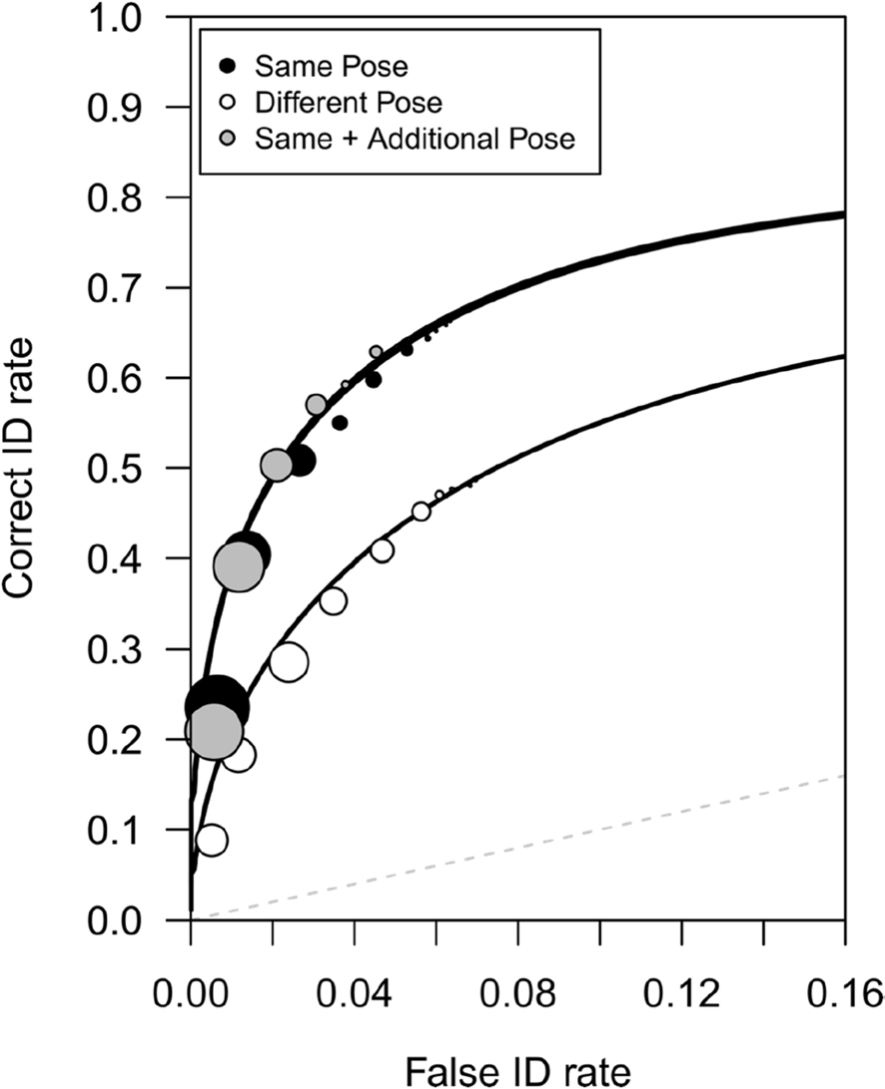
Experiment 1 ROC data in the same pose, different pose, and same + additional pose conditions. The circles are the empirical data and the curved lines of best fit were generated using the Independent Observations model. The bottom x-axis shows the estimated false ID rate of innocent suspects. The dashed line indicates chance-level performance. The size of the symbols represents the number of suspect IDs at a given level of confidence relative to the total number of suspect IDs collapsed across all levels of confidence^31^.

We conducted ROC analysis to measure participants’ collective ability to discriminate between perpetrators and innocent suspects. Figure 2 shows the ROC curves for the same-pose, different-pose, and same + additional-pose conditions (see Mickes et al., 2012, for a tutorial)^23^. It is apparent that those in the same-pose and same + additional pose conditions discriminated perpetrators from innocent suspects better than those in the different-pose condition. Partial area under the curve (*p*AUC) values were computed using a target-absent filler ID cut-off (i.e., specificity) of 0.618 with the statistical package pROC^30^. We used a Bonferroni-corrected alpha level of 0.017. As predicted by the pose-reinstatement hypothesis, the *p*AUC for the same-pose condition (0.186) was significantly greater than the *p*AUC for the different-pose condition (0.117), *D* = 5.43, *p* < 0.001. The *p*AUC for the same + additional pose condition (0.194) was also greater than the *p*AUC for the different-pose condition (0.117), *D* = 6.29, *p* < 0.001. Although the same + additional pose condition yielded a slightly higher *p*AUC (0.194) than the same-pose condition (0.186), that difference was not statistically significant, *D* = 0.639, *p* = 0.523.

We conducted additional ROC analyses to study pose reinstatement effects in each perpetrator encoding pose condition (see Supplementary Appendix A). The results indicated that discrimination accuracy was higher when the lineup members could be seen in the same pose compared to when they were seen in a different pose in all encoding conditions, albeit the effect was more reliable in the frontal pose than in the right-profile condition. The increase in discrimination accuracy appears larger in the frontal compared to the profile encoding condition (see Supplementary Fig. A1).

We also fit a signal-detection model to the data which accounts for all identification decisions (see Supplementary Appendix B) and the results agreed with the *p*AUC results reported here. Compared to when participants had different pose information at test (*d*′ = 1.67), discrimination accuracy was better when they had the same pose information that they encoded (*d*′ = 2.17; χ^2^ (1) = 35.37, *p* < 0.001) and when they had the same pose information plus additional unstudied information (*d*′ = 2.02; χ^2^ (1) = 39.51, *p* < 0.001). Discrimination accuracy was not boosted further when participants had available at test additional unstudied information about the face (*d*′ = 2.20; χ^2^ (1) = 0.10, *p* = 0.752). The same was true when the model was fit to each perpetrator encoding pose condition separately, and the effect appears larger in the frontal compared to the profile encoding condition (see Supplementary Appendix B). Taken together, these supplementary results support the *pose-reinstatement hypothesis*.

Analyses of the confidence accuracy relationship by lineup member pose condition are presented in Supplementary Appendix C.

In sum, the results of Experiment 1 support the pose-reinstatement hypothesis: consistent viewpoint information at study and at test improved lineup discrimination accuracy. These findings conceptually replicate face recognition research using an episodic memory paradigm and an eyewitness identification task.

## Experiment 2

In Experiment 2, we extended Experiment 1 by examining whether participants would *actively seek* to reinstate during a lineup test the pose in which they had encoded a perpetrator commit a mock crime (i.e., left-profile or right-profile). Relatedly, an interactive virtual reality eyewitness memory study with avatars reported that having multiple face viewpoints available at test increased accuracy, but only when the perpetrator was present in the lineup^32^. However, this study was likely underpowered, and did not vary encoding pose or measure discrimination accuracy. We developed an interactive lineup procedure that enabled participants to rotate the lineup faces into any pose desired. The procedure recorded the length of time participants spend viewing the faces at different angles. We predicted that participants would naturally reinstate the lineup faces, particularly the perpetrator’s face, into the same pose as they had encoded the perpetrator, and that greater pose reinstatement would be associated with better discrimination accuracy. We pre-registered our hypotheses and analysis plan before we collected data (https://osf.io/ezsxg).

### Design

We used a 2 (perpetrator encoding pose: left-profile, right-profile) × 2 (target: present, absent) between-subjects design.

### Participants

Participants (*N* = 1727) were recruited from Amazon Mechanical Turk; they were remunerated 0.45 cents. Participants who incorrectly answered the attention check question (*n* = 40) and those who did not interact with any of the faces in the lineup (*n* = 257) were excluded, leaving a total of 1430 participants (56% female, 43% male, 1% preferred not to say or stated ‘other’; 18–83 years old, *M* = 36.93, *SD* = 12.06 years; 75% Caucasian, 9% Black or African American, 6% Hispanic, 6% East Asian, 3% South Asian, 1% American Indian or Alaska Native, < 1% Native Hawaiian or other Pacific Islander, and 0% Other ethnicities/races or prefer not to say). None of the participants who took part in Experiment 1 took part in Experiment 2.

Of the 1430 participants who were included in the data analysis, 725 viewed the right-profile mock-crime video (352 participants were assigned a target-present lineup and 373 were assigned a target-absent lineup) and 705 viewed the left-profile crime video (355 were assigned a target-present lineup and 350 were assigned a target-absent lineup). Participants were randomly allocated into conditions, with the constraint that the number of participants in each condition was relatively equal. Our data-collection stopping rule was to recruit 1400 participants—350 in each of the between-subject conditions. Using the medium effect sizes determined in a pilot study, a power analysis indicated that, with 350 subjects per between-subjects condition, power would exceed 80% in all of our planned analyses.

### Materials

We used the same mock crime as Experiment 1, but this time we used only the right-profile and left-profile crime videos. Unlike Experiment 1, participants could interact with the lineup members and view them from any angle desired. To this end, we took images of the lineup members and rendered them into rotatable objects using our *Eyewitness Interactive* program. The lineup members were the same as those used in Experiment 1, but this time we used 6 lineup members, instead of 9, for use in our Eyewitness Interactive software. Target-present lineups contained the perpetrator and 5 fillers, target-absent lineups contained 6 fillers. To assess perceptual fairness in the 6-person interactive lineups, we conducted a mock-witness test and presented a new group of participants (*N* = 50) with the description of the perpetrator followed by the target-present lineup or target-absent lineup. The perpetrator was identified 12% of the time in the target-present lineup and the most frequently chosen lineup member was identified 25% of the time in the target-absent lineup; these rates did not significantly exceed chance expectation (17%), *p* > 0.05. Effective size was 5.63 (95% CI [4.78, 6.86]) in the target-present lineup and 5.05 (95% CI [4.00, 6.86]) in the target-absent lineup. This means that there were approximately 5 members who were viable alternatives from which the witness might choose. Together, the mock-witness test illustrated that our lineups were perceptually fair, based on the description of the perpetrator.

### Procedure

We used the same procedures as in Experiment 1 with three exceptions. First, participants either saw the left-profile or right-profile mock-crime video. Second, to reduce the overall length of the experiment, we removed the anagrams, so participants just watched the distractor cartoon for 1 min and 11 s during the filler task. Finally, at test, we used an interactive lineup. During the interactive lineup, each lineup member was initially shown in frontal pose and participants were instructed that they could use their mouse or touchpad to rotate the face so that they could view it from other angles. If participants did not interact, the face was shown facing forward the entire time. The program used to present the interactive lineup recorded whether, and for how long, participants examined five regions of the face (left-profile, left three-quarter, frontal, right three-quarter, and right-profile). It is important to note that we were interested in how participants behaved naturally. As such, we did not constrain the length of time participants spent on each of the members to be equal, and we did not force participants to interact with the faces in any particular manner. We did, however, encourage participants to interact. Before the lineup, we told participants: “*We want to see how people interact with these faces. We are recording what you are doing with your mouse*.” Participants had to view each lineup member for at least 5 s, after which time, they could press the “next” button to proceed to the next face or, if they had already seen all 6 faces, make a lineup decision. As such, the stopping rule was that participants had to see every lineup face before making a decision. Under each lineup member, we reminded participants: “*Remember to click on and rotate the face. Please examine the face for as long as you need. You must wait 5 s before you can continue*”.

## Results

Our data are available (https://osf.io/vtmdb/). Response frequencies for perpetrator, filler, and reject decisions at every level of confidence for the right-profile and left-profile encoding conditions are displayed in Table 3. As in Experiment 1, the false ID rate was estimated by dividing the number of target-absent filler IDs by the number of lineup members (i.e., 6 in this experiment). That estimated value was then divided by the number of target-absent lineups to estimate the false ID rate. The overall correct ID rates were 0.67 and 0.74 for the left-profile and right-profile encoding conditions, respectively. The corresponding false ID rates were both 0.07 for the left-profile (0.41/6) and the right-profile (0.43/6) conditions.

**Table 3:** Frequencies of perpetrator, filler and reject identification decisions in interactive lineups by encoding condition in Experiment 2.

Confidence rating	Left-encoding					Right-encoding				
	Target-present			Target-absent		Target-present			Target-absent	
	Perp	Filler	Reject	Filler	Reject	Perp	Filler	Reject	Filler	Reject
0	1	0	2	0	1	0	0	0	0	0
10	1	1	0	1	0	1	1	1	2	1
20	1	2	0	2	0	1	1	1	4	1
30	3	1	0	5	3	1	2	2	6	7
40	7	1	4	9	1	2	5	1	6	6
50	14	4	9	24	17	9	6	4	21	7
60	16	6	6	27	10	14	12	1	27	17
70	35	16	13	22	30	38	7	6	39	31
80	48	4	21	29	54	46	12	10	33	44
90	53	5	11	11	39	67	8	5	15	47
100	58	1	11	13	52	80	4	4	8	51
Total	237	41	77	143	207	259	58	35	161	212
Proportion	0.67	0.12	0.22	0.41	0.59	0.74	0.16	0.10	0.43	0.57

Given the observed differences in the correct ID rates, we conducted ROC analysis to see whether discrimination accuracy was significantly different between left- and right-profile encoding conditions. Note that this analysis was not part of our pre-registered plan because we did not expect to see any differences between the left- and right-profile encoding conditions. Those assigned to the right-profile condition were slightly better at discriminating innocent from guilty suspects than those assigned to the left-profile condition. The *p*AUC (computed using a target-absent filler ID cut-off of 0.59) for the right-profile condition (0.228) was significantly greater than the *p*AUC for the left-profile condition (0.191), *D* = 2.35, *p* = 0.019. The results of a signal-detection model fit to the data partially agreed with the *p*AUC results reported here: *d*′ was estimated to be higher in the right-profile (2.14) than the left-profile (1.95) encoding condition, but that difference did not quite reach statistical significance, χ^2^ (1) = 3.55, *p* = 0.060. We had not expected to see any differences in accuracy between the left- and right- profile encoding conditions, but previous research has found that the right compared to the left side of a face is perceptually more salient to an observer^33^, which may explain why participants were more accurate when they encoded the perpetrator in right-profile pose. Given the unexpected difference in accuracy between the right-profile and left-profile, in our subsequent analyses, we ran additional tests to check that the results were consistent across both the left- and right- encoding conditions.

### Perpetrator pose reinstatement

We hypothesized that participants would turn the lineup faces to reinstate the same pose as they had viewed the perpetrator in the mock-crime video, and also hypothesised that this pose-restatement effect would be larger for the perpetrator’s face compared to filler faces in the target-present lineup. To measure pose reinstatement, we calculated for every participant and lineup face the proportion of time that the participant spent examining the same profile side of the lineup face as the perpetrator had been shown, as well as the proportion of time that participants spent examining the opposite profile of the lineup face relative to the profile view in which the perpetrator had been shown. The proportion of time spent on the same profile of the face was measured by determining the total amount of time a lineup face was rotated and paused within the same profile region divided by the total length of time that the lineup face was on screen. Proportion of time spent on the opposite profile of the face was similarly determined, summing the total length of time the lineup face was rotated and paused within the opposite profile region and dividing by the total length of time that the lineup face was on screen. To examine how the subject interacted with the target compared to the filler faces, we averaged across the filler faces the same side proportion data and the opposite side proportion data.

**Figure 3:**
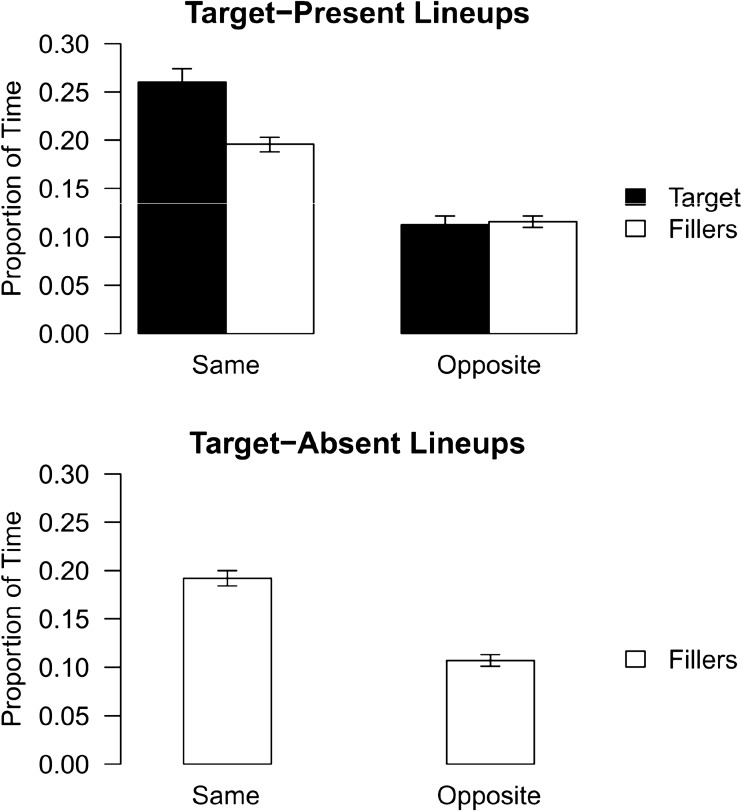
Mean (+ 1SEM) proportion of time participants spent in the target-present and target-absent lineup conditions viewing the perpetrator and filler lineup faces in the same profile compared to the opposite profile as the perpetrator’s face had been shown in the crime video.

To examine pose reinstatement in target-present lineups, we conducted a 2 (perpetrator pose reinstatement: same, opposite) × 2 (face type: perpetrator, filler) repeated measures ANOVA using the proportion of time data from the target-present condition. The top panel of Fig. 3 shows the target-present lineup viewing time data plotted by encoding condition. As predicted, participants spent a larger proportion of time examining the lineup faces in the same profile as they had encoded the perpetrator compared to the opposite profile (*M* = 0.23, SEM = 0.005 versus *M* = 0.11, SEM = 0.003, respectively), a large sized significant main effect for pose reinstatement, *F*(1, 670) = 425.39, *p* < 0.001, η_p_^2^ = 0.39. Participants also spent a larger proportion of time examining the perpetrator’s face compared to the filler faces in profile view (*M* = 0.19, *SEM* = 0.004 versus *M* = 0.16, *SEM* = 0.003, respectively), a moderately sized significant main effect for face type, *F*(1, 670) = 84.92, *p* < 0.001, η_p_^2^ = 0.11. As we had hypothesized, there was also a pose reinstatement × face type interaction effect, *F*(1, 670) = 58.32, *p* < 0.0001, η_p_^2^ = 0.08. Participants apportioned more time viewing the perpetrator’s face in the same profile as the perpetrator had been shown compared to the fillers’ faces (*M* = 0.26, *SEM* = 0.007 versus *M* = 0.20, *SEM* = 0.004), *t*(670) = 9.76, *p* < 0.001. The proportion of time spent viewing the profile of the faces that was opposite to that in which the perpetrator had been shown did not differ by face type (perpetrator *M* = 0.11, *SEM* = 0.004 versus filler *M* = 0.12, *SEM* = 0.003), *t*(680) = − 0.256, *p* > 0.05.

To examine pose reinstatement in target-absent lineups, we conducted a 2 (perpetrator pose reinstatement: same, opposite) repeated measures ANOVA using the proportion of time data from the target-absent condition. Descriptive data are shown in the bottom panel of Fig. 3. As predicted, participants spent a larger proportion of time viewing the lineup faces in the same profile as they had encoded the perpetrator compared to the opposite profile (*M* = 0.19, *SEM* = 0.004 versus *M* = 0.11, *SEM* = 0.003, respectively), a large sized significant main effect for pose reinstatement, *F*(1, 720) = 298.5, *p* < 0.001, η_p_^2^ = 0.29.

There are two things to note about the ANOVA results reported above in relation to our data analysis plan reported in the OSF preregistration (https://osf.io/ezsxg). First, as per the preregistration, we did not include encoding condition as a variable in the ANOVAs. However, since we unexpectedly found higher discrimination accuracy in the right- compared to the left-profile condition in our preliminary examination of the data, we ran an additional test and checked whether encoding condition had an effect on the ANOVA results reported here. When encoding condition is included in the model, the pattern and statistical significance of the results we report here do not change. Second, as stated in our OSF preregistration, we had planned to include accuracy (correct versus incorrect ID decision) in the ANOVA. However, given that this treatment of accuracy would confound discrimination accuracy with response bias^23^, we instead examined the association between pose reinstatement and discrimination accuracy using ROC analysis. We present these analyses next.

### Pose reinstatement and ID discrimination accuracy

We investigated the relationship between pose reinstatement and discrimination accuracy. We predicted that greater pose reinstatement would be associated with better discrimination accuracy. To test this prediction, we measured the proportion of time participants reinstated the lineup faces into the same pose in which the perpetrator had been seen in the mock-crime video (collapsed across target-presence and lineup member type). We created two pose-reinstatement groups: high and low pose reinstatement, using a median split. The overall correct and false ID rates for the high pose-reinstatement group were 0.73 and 0.10, respectively. Whereas the overall correct and false ID rates for the low pose-reinstatement group were 0.67 and 0.10, respectively.

#### ROC analysis

**Figure 4:**
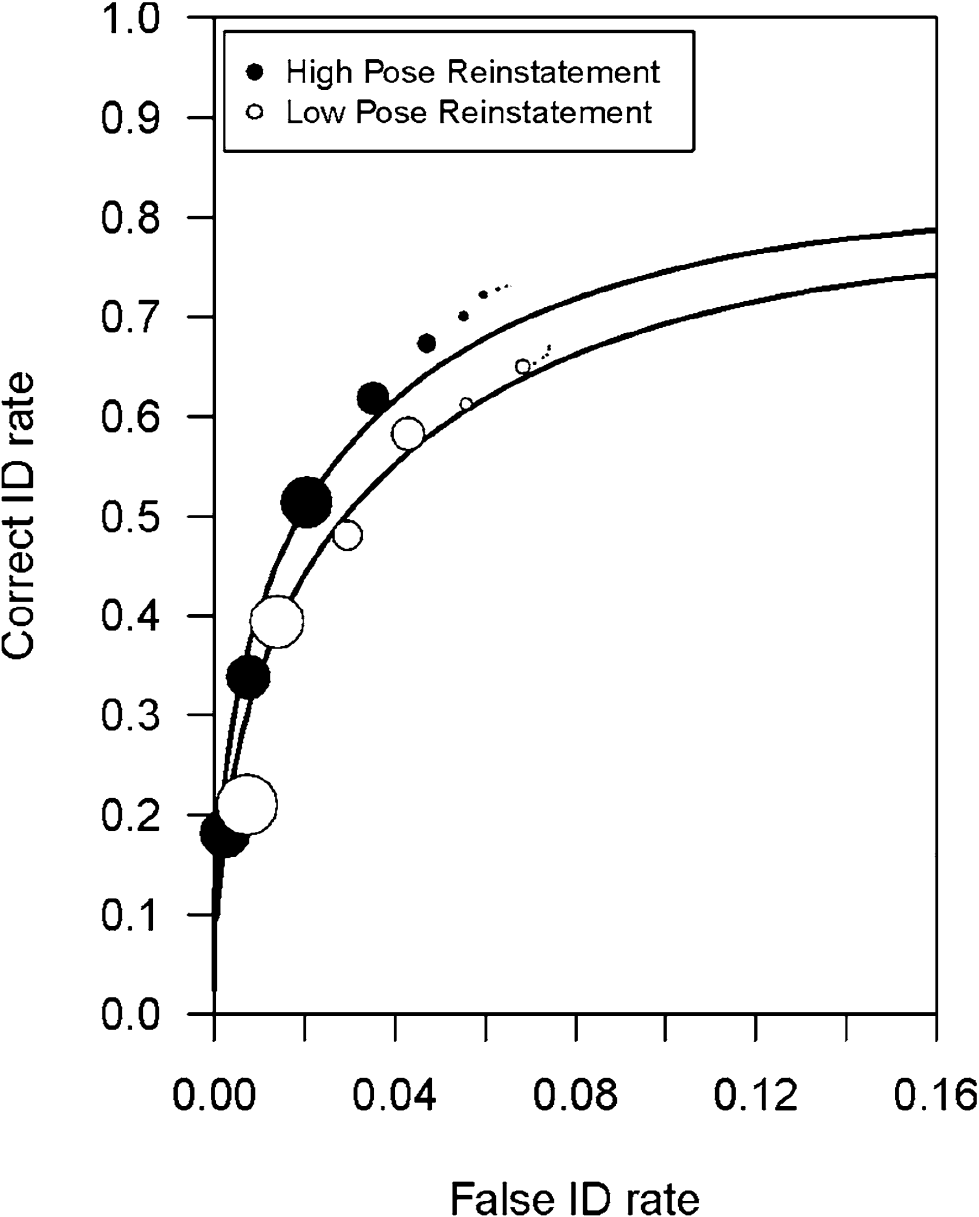
Experiment 2 ROC data for the high pose-reinstatement and low pose-reinstatement participants. In (**A**), the circles are the empirical data and the curved lines of best fit were generated using the Independent Observations model. The bottom x-axis shows the estimated false ID rate of innocent suspects. The dashed line indicates chance-level performance. The size of the symbols represents the number of suspect IDs at a given level of confidence relative to the total number of suspect IDs collapsed across all levels of confidence (Seale-Carlisle et al., 2019)^34^.

To see whether discrimination accuracy is significantly greater for those in the high pose-reinstatement group, we conducted ROC analysis; Fig. 4 shows these ROCs. The high pose-reinstatement group yielded a significantly greater *p*AUC (0.217) than the low pose-reinstatement group (0.184), *D* = 2.148, *p* = 0.03. Note that a similar pattern of results was found across the encoding conditions. In the right-profile encoding condition, there was a non-significant trend for the *p*AUC to be greater in the high (0.233) compared to low (0.196) pose-reinstatement group, *D* = 1.78, *p* = 0.08. In the left-profile condition, there was also a non-significant trend for the *p*AUC to be greater in the high (0.199) compared to the low (0.176) pose-reinstatement group, *D* = 1.01, *p* = 0.31. Again, when we fit a signal-detection model to the data (Supplementary Appendix B), the results agreed with the *p*AUC results reported here: *d*′ was estimated to be greater for the high pose-reinstatement group (2.14) than the low pose-reinstatement group (1.97), though that difference did not reach statistical significance, χ^2^ (1) = 3.04, *p* = 0.08.

Analyses of the confidence-accuracy relationship for the low and high pose reinstatement groups are presented in Supplementary Appendix C.

## General discussion

We investigated whether pose-reinstatement effects, which have been demonstrated only with standard face recognition paradigms, generalize to the eyewitness identification context. As predicted by the encoding specificity principle, in two experiments, we found that accuracy varied in relation to whether the participant had the same or different pose information in the lineup task as was encoded during the mock crime. Notably, our novel interactive lineup test, which allowed participants to rotate and view the lineup members in any angle, enabled us to test whether participant witnesses naturally seek to reinstate perpetrator pose at test, and if pose reinstatement was associated with discrimination accuracy. We found that participants reinstated perpetrator pose during the interactive lineup task and high pose reinstatement was associated with accuracy.

Consistent with the basic face recognition literature^8, 10^, our results show that people store viewpoint information in memory, even when face learning occurs under more naturalistic conditions. Remarkably, without instruction, participants spontaneously rotated the lineup faces to match the pose in which they had encoded the perpetrator. This suggests that people—consciously or unconsciously—value the information that can be gleaned from reinstating pose when making an identification decision from a lineup. More broadly, it evidences that humans actively seek out information in the testing environment that matches the study environment to help them to retrieve information from memory, a result that is in line with other eyewitness research varying the overlap between cues at learning and at test^14–17^.

Our study provides evidence that identification procedures allowing participants to reinstate perpetrator pose could boost discrimination accuracy. Research comparing the efficacy of static frontal pose photo lineups and video lineup procedures has found mixed results^35–39^. Seale-Carlisle et al.^31^ also compared photo and video lineups and found no significant difference (though photo lineups yielded a higher ROC). However, perpetrator encoding angle, and the overlap between the angle of the faces at encoding and test was not considered in any of these studies. Our data suggest that showing the lineup members in the same pose that the perpetrator was viewed, or conducting interactive lineups—that allow witnesses to actively seek the pose information they require—could significantly increase eyewitness discrimination accuracy compared to existing lineup procedures. A recent meta-analysis of the literature^31^ indicated that the weighted average pAUC for sequentially presented static frontal pose photographic lineups is 0.100% CI [0.081, 0.120], which, descriptively speaking, is lower than the pAUCs (left encoding pAUC = 0.198 and right encoding pAUC = 0.221) we obtained for interactive lineups in the present study. Further, we found in two experiments, one conducted in lab and the other online, that interactive lineups boost discrimination accuracy over static frontal pose photographic lineups, increasing the correct identification of guilty suspects by 18%^40^.

Considering our main findings, and the limitations of this study, we suggest five key areas for further research. First, future research could systematically control the rotation of the lineup faces and assess the effect on accuracy, particularly using a wider variety of encoding poses and significantly longer retention intervals. The benefit of pose reinstatement may vary depending on the difficulty of encoding. Discrimination accuracy was higher when the perpetrator was studied in frontal as opposed to profile view. When participants encoded the perpetrator from front-on, discrimination accuracy was higher when the frontal pose was available at test compared to when it was not available. While there was also a pose reinstatement effect when the perpetrator was encoded in profile view, the size of the effect was smaller. Second, it would be interesting to examine the role of movement as a recognition cue in the context of lineups. According to the representation enhancement hypothesis, facial motion (i.e., rigid motion, such as turning the head, and elastic motion, such as smiling) aids perception 3D structure^41, 42^. Third, another key direction for further research is to use interactive lineups as a tool to study memory retrieval processes, such as whether people search for diagnostic versus non-diagnostic features^43^. The interactive procedure allows for exploring face viewing behaviour in online studies with large samples of participants, and therefore, is a useful adjunct to other methods being used to study decision processes, such as eye tracking^44^ and response time^34, 45, 46^. Fourth, like every lineup procedure, the interactive lineup comprises different components (e.g., movement, multiple viewing angles, interactivity, sequential viewing). One component or a combination of components may be responsible for improvements in eyewitness accuracy. We have demonstrated that interactive lineups can facilitate pose reinstatement and improve accuracy and additional research should determine which components of the procedure have the largest effect on accuracy. Finally, future work is needed to ascertain whether eyewitnesses are able to accurately report the pose in which they encoded the perpetrator, and whether doing so is necessary for obtaining pose reinstatement effects. In the case of the infamous serial killer Ted Bundy, for example, a lineup identification was called into question by the defense owing to differences between encoding and test pose^47^.

In sum, our findings provide evidence that allowing witnesses to reinstate pose at test increases discrimination accuracy. Our results deepen our theoretical understanding of how faces are processed during learning and remembering, particularly in more naturalistic contexts. Our work follows the example of others in innovating lineup procedures^48–50^ and answers the National Academy of Sciences’^3^ call for the use of psychological theory and innovative technology to improve the accuracy of eyewitness identification.

## Supplementary Information

Supplementary Information.

## Data Availability

Our data are freely available (Experiment 1: https://osf.io/jm2k9/ and Experiment 2: https://osf.io/vtmdb/).
