# Supplementary material for: Perpetrator pose reinstatement during a lineup test increases discrimination accuracy
Source: Sci Rep. 2021 Jul 9;11:13830. doi: 10.1038/s41598-021-92509-0 (PMC8271008; doi:10.1038/s41598-021-92509-0)
Supplement: Supplementary file 1 — Supplementary Information. [file 41598_2021_92509_MOESM1_ESM.docx]

Perpetrator pose reinstatement during a lineup test increases discrimination accuracy

Melissa F. Colloff^1^, Travis M. Seale-Carlisle^1,2^, Nilda Karoğlu^3^, James C. Rockey^4^, Harriet M. J. Smith^5^, Lisa Smith^6^, John Maltby^7^, Sergii Yaremenko^8^, & Heather D. Flowe^1*^

University of Birmingham, Centre for Applied Psychology, School of Psychology^1^

Duke University, Center for Science and Justice, School of Law^2^

University of Kent, School of Psychology^3^

University of Birmingham, Department of Economics^4^

Nottingham Trent University, Division of Psychology^5^

University of Leicester, Department of Criminology^6^

University of Leicester, School of Psychology^7^

Maastricht University, Department of Clinical Psychological Science^8^

**Appendix A**

**ROC Analysis to Test Pose Reinstatement Hypothesis within each Perpetrator Encoding Pose Condition**

We conducted ROC analysis to measure participants’ collective ability to discriminate between perpetrators and innocent suspects in the same-pose, different-pose, and same + additional-pose conditions for the right-profile and the front perpetrator encoding conditions in Experiment 1 (see Figure A1). The partial area under the curve (*p*AUC) values were computed using with the statistical package pROC.^1^ We used a target-absent filler ID cut-off (i.e., specificity) of .72, which was based on the smallest false alarm rate to any filler face obtained across the six experimental conditions (i.e., .28, front perpetrator encoding condition, same + additional pose lineup condition). Within each perpetrator encoding pose condition, as we had predicted, the *p*AUCs obtained for the same-pose and same + additional pose conditions are larger compared to the *p*AUC obtained for different-pose condition, suggesting discrimination accuracy was higher when the lineup members could be seen in the same pose as the perpetrator had been encoded (see Figure A2).


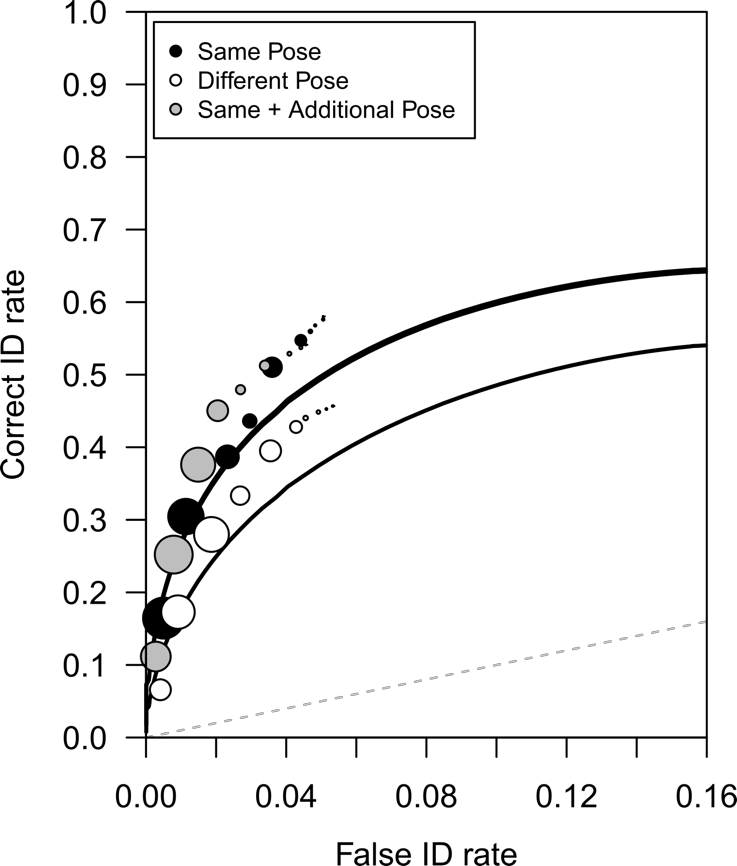

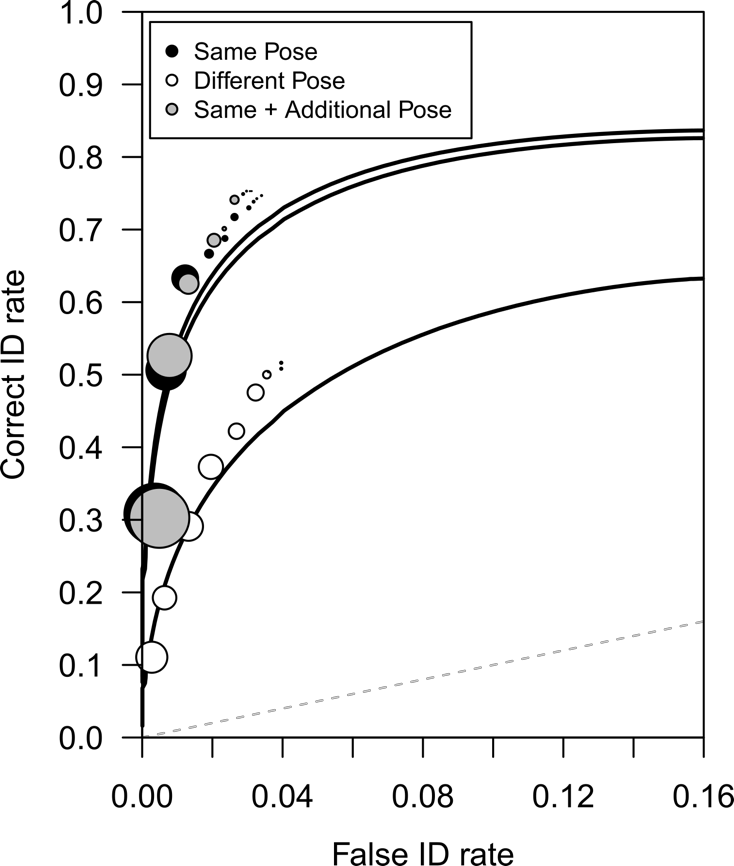
A B

*Figure A1. Experiment 1 ROC data in the same pose, different pose, and same + additional pose conditions within the frontal (Panel A) and right-profile (panel B) encoding conditions. The circles are the empirical data and the curved lines of best fit were generated using the Independent Observations model reported in Appendix B. The bottom x-axis shows the estimated false ID rate of innocent suspects. The dashed line indicates chance-level performance. The size of the symbols represents the number of suspect IDs at a given level of confidence relative to the total number of suspect IDs collapsed across all levels of confidence.*


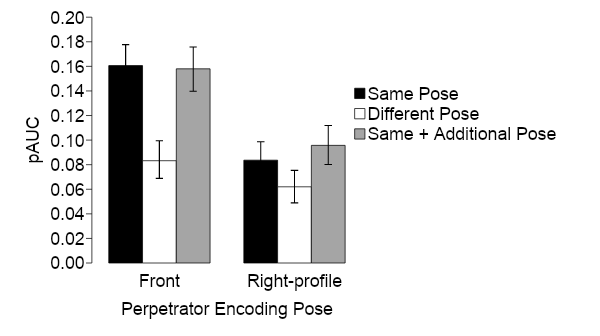


*Figure A2*. Partial area under the curves (pAUCs) for each lineup member pose condition for the front and the right-profile encoding pose conditions. The error bars represent the 90% confidence intervals.

We statistically compared the *p*AUCs using the proportion of overlap (POL) between the CIs inference-by-eye method^2^, which compares the lower arm of the CI of the highest mean with the upper arm of the CI of the lowest mean. For a directional hypothesis test and an alpha level of .05, the 90% CI is calculated for each *p*AUC. If the POL is less than .30, the difference between the *p*AUCs is significant at *p* < .05 (Cumming, 2008). (Contrary to our pre-registration, we used the 90% rather than 95% confidence intervals, because the 90% interval should be calculated to test the one-tailed hypotheses.) In the front perpetrator encoding condition, the *p*AUCs for same-pose condition (*p*AUC = .16, 90% CI [.14, .18]) and the same + additional pose condition (*p*AUC = .16, 90% CI [.14, .18]) were significantly greater than the *p*AUC for the different-pose condition (*p*AUC = .08, 90% CI [.07, .10]). The *p*AUCs for the same-pose and the same + additional pose conditions did not significantly differ (.16 versus .16). In the right-profile perpetrator encoding condition, the *p*AUC for the same + additional pose information condition (*p*AUC = .10, 90% CI [.08, .11]) was significantly higher than for the different-pose condition (*p*AUC = .06, 90% CI [.05, .08]). Although the same-pose condition yielded a higher *p*AUC (.08, 90% CI [.07, .10]) than the different-pose condition (.06, 90% CI [.05, .08]), that difference was not statistically significant. However, it is likely that the non-significant result was due to the small false alarm rate restricting the pAUC calculation to a portion of the ROCs in this analysis. With a target-absent filler ID cut-off (i.e., specificity) of .54, which was based on the smallest false alarm rate to any filler face obtained across these two experimental conditions (i.e., .46) that are being compared, the *p*AUC for the same-pose condition was significantly higher compared to the *p*AUC for the different-pose condition (.18 90% CI [.16, .20] versus .14 90% CI [.12, .16], respectively).

**Appendix B**

**Signal Detection Model-fit Analysis to Test Pose Reinstatement Hypothesis**

We fit an equal variance signal-detection model to our data; assuming an independent observations decision rule (see Wixted, Vul, Mickes, & Wilson, 2018, for a description of the independent observations rule and other signal-detection models for eyewitness data)^3^. The benefit of fitting a model is that it accounts for all identification decisions (perpetrator, target-present and target-absent fillers, and lineup rejections) and measures underlying theoretical discriminability *d*′, which is the ability to discriminate between faces that have (i.e., the perpetrator) and have not been seen before. In practice, the results of an atheoretical *p*AUC analysis often correspond with the results from a theoretical model-fit analysis. Nevertheless, there are times where findings can diverge, so we checked that here. Table B1 reports the model-fit parameters and fits for Experiment 1 and 2. We estimated *d*′, and criterions 1-3, which reflect different levels of confidence (c_1_: 0-60% certain, c_2_: 70-80% certain, c_3_: 90-100% certain). The fit of the model to the data can be examined using the Σχ^2^, *df* and *p* values for each model fit. Although the model predicted values significantly deviated from the observed values in two fits in Table B1 (i.e., model fits where *p* = .035 and *p* = .016), we used the model-predicted values to draw the lines of best fit through the empirical data points in Figures 2 and 4 and Figure A1. Although a more complex model may fit the data better, this simple model can account for the qualitative patterns observed in the data.

| Table B1  *Model parameters and fits using the independent observations model* | | | | | | | | | |
| --- | --- | --- | --- | --- | --- | --- | --- | --- | --- |
| Experiment | Condition | Parameters and model-fit statistics | | | | | | | |
|  |  | *d*′ | c_1_ | c_2_ | c_3_ | χ^2^ | Σ χ^2^ | *df* | *p* |
| 1 | Same | 2.17 | 1.62 | 1.97 | 2.40 | 5.39 | 26.25 | 15 | .035 |
|  | Different | 1.67 | 1.62 | 2.02 | 2.51 | 17.27 |  |  |  |
|  | Same + additional | 2.20 | 1.67 | 1.99 | 2.45 | 3.59 |  |  |  |
| 1: Front Encoding | Same | 2.53 | 1.77 | 2.07 | 2.51 | 2.89 |  |  |  |
|  | Different | 1.80 | 1.72 | 2.10 | 2.59 | 9.94 | 15.66 | 15 | .405 |
|  | Same + additional | 2.56 | 1.80 | 2.05 | 2.47 | 2.83 |  |  |  |
| 1: Profile Encoding | Same | 1.85 | 1.49 | 1.89 | 2.31 | 8.70 |  |  |  |
|  | Different | 1.53 | 1.52 | 1.96 | 2.44 | 10.72 | 22.53 | 15 | .095 |
|  | Same + additional | 1.85 | 1.56 | 1.95 | 2.46 | 5.13 |  |  |  |
| 2 | Right profile | 2.13 | 1.30 | 1.69 | 2.33 | 11.44 | 21.86 | 10 | .016 |
|  | Left profile | 1.94 | 1.42 | 1.81 | 2.40 | 10.42 |  |  |  |
| 2 | High pose- reinstatement | 2.14 | 1.40 | 1.82 | 2.54 | 8.70 | 9.72 | 10 | .465 |
|  | Low pose- reinstatement | 1.96 | 1.34 | 1.68 | 2.22 | 1.02 |  |  |  |

We conducted additional model-fitting to study pose reinstatement effects in each perpetrator encoding pose condition. The results were the same as the results reported in the main paper, where we collapsed over perpetrator encoding pose. In the front encoding condition, compared to when participants had different pose information at test (*d*′ = 1.80), discrimination accuracy was better when they had the same pose information that they encoded (*d*′ = 2.53; χ^2^ (1) = 34.05, *p* < .001) and when they had the same pose information plus additional unstudied information (*d*′ = 2.56; χ^2^ (1) = 37.13, *p* < .001). Discrimination accuracy was not boosted further when participants had available at test additional unstudied information about the face (χ^2^ (1) = 0.05, *p* = .825). Similarly, in the profile encoding condition, compared to when participants had different pose information at test (*d*′ = 1.53), discrimination accuracy was better when they had the same pose information that they encoded (*d*′ = 1.85; χ^2^ (1) = 7.14, *p* = .008) and when they had the same pose information plus additional unstudied information (*d*′ = 1.85; χ^2^ (1) = 7.03, *p* = .008). Discrimination accuracy was not boosted further when participants had available at test additional unstudied information about the face (χ^2^ (1) = 0.00, *p* = .993).

**Appendix C**

**Analysis of the Confidence-Accuracy Relationship**

***Experiment 1***

Figure C1 shows the confidence-accuracy relationships for the same-pose, different-pose, and same + additional pose conditions Experiment 1, with the data collapsed across encoding condition. The relationship between confidence and accuracy was analysed using confidence accuracy characteristic (CAC) curves, which measure the probability that a suspect who has been identified is guilty (also known as proportion correct or positive predictive value) at different confidence levels. CAC analysis only includes suspect IDs, and thus the CAC dependent variable = (correct suspect ID rate) / (correct suspect ID rate + ~ incorrect suspect ID rate) for every level of confidence, where correct ID rate is the number of guilty suspect IDs / number of target present lineups, and the where ~incorrect suspect ID rate refers to estimated innocent suspect ID rate obtained by dividing filler IDs from target-absent lineups by lineup size (which is standard practice in the field for estimating the number of innocent suspect IDs from fair target-absent lineups), and then diving that by the number of target absent lineups. We binned the data into low (ratings of 0-60), medium (ratings of 70-80) and high (ratings of 90-100) levels of confidence because there were too few suspect IDs in certain bins, especially at the lowest levels of confidence, in line with previous work.^4^ The sizes of the symbols in the figure represents the number of suspect IDs at a given level of confidence bin relative to the number of IDs given at other levels of confidence bins. The standard error bars for the suspect ID accuracy scores shown in Figure C1 were estimated using a bootstrap procedure. Figure C1 shows that, within each lineup member pose condition, IDs made with high confidence and medium confidence were higher in accuracy than identifications made with low confidence. The pattern of results does not generally vary reliably across lineup member pose condition, as indicated by the overlap in the standard error bars. Figure C2 provides CACs for the frontal pose encoding condition (Panel A) and for the profile pose encoding condition (Panel B). Again, the pattern of results does not generally vary across lineup member pose condition.


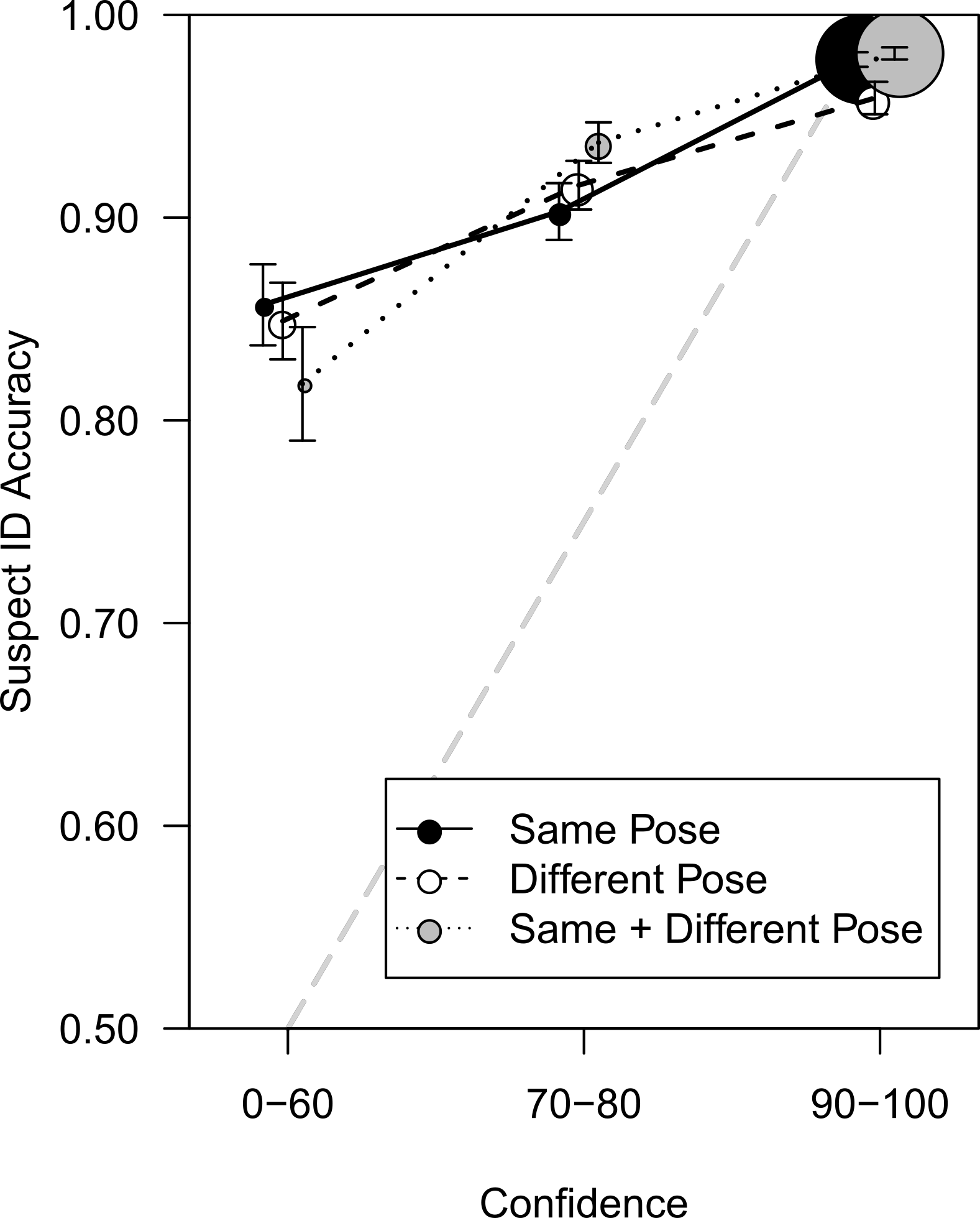


*Figure C1*. Confidence accuracy characteristic curves (CACs) for each lineup member pose condition, collapsed across encoding condition. The size of the symbols represents the number of suspect IDs at a given level of confidence relative to the total number of suspect IDs collapsed across all levels of confidence. Error bars reflect +1 SE.

*
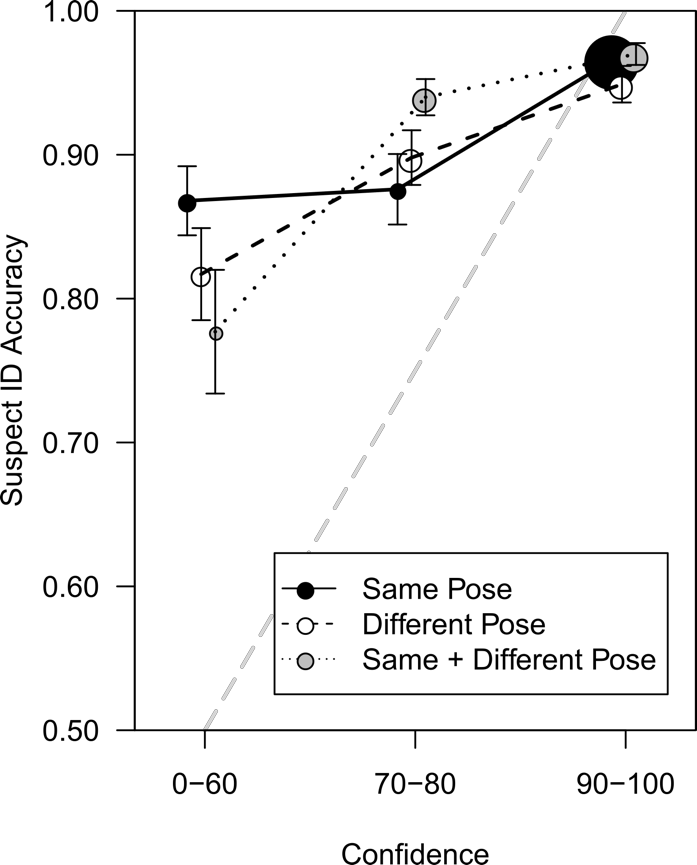
*
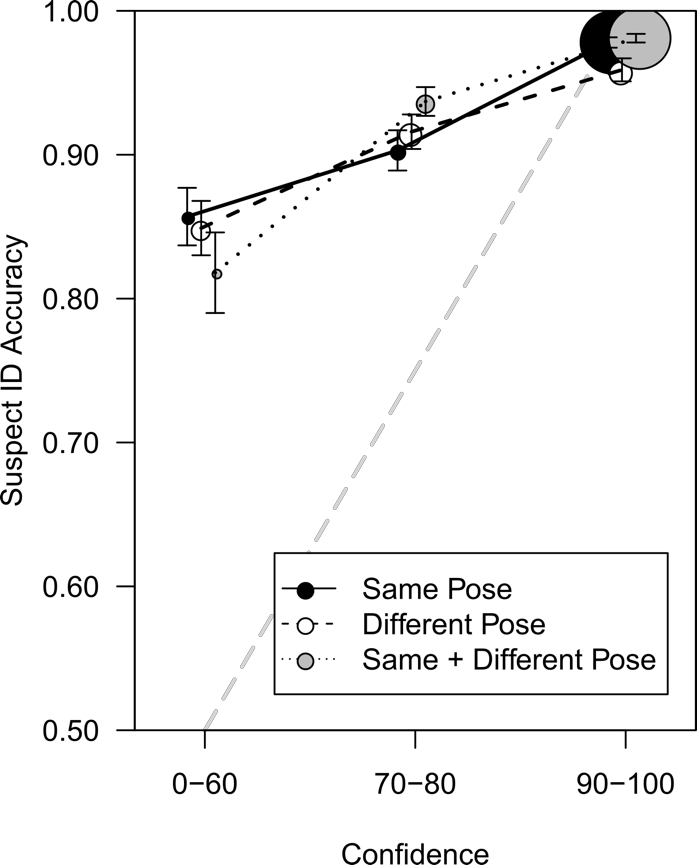
A B

*Figure C2*. Confidence accuracy characteristic curves (CACs) for each lineup member pose condition in the frontal pose encoding condition (Panel A) and the profile pose encoding condition (Panel B). The size of the symbols represents the number of suspect IDs at a given level of confidence relative to the total number of suspect IDs collapsed across all levels of confidence. Error bars reflect +1 SE.

Calibration statistics can provide further information about the confidence-accuracy relationship. The calibration statistic (C) reflects the level of deviation from perfect calibration, with 0 corresponding to perfect calibration and 1 to poorest calibration. The over/underconfidence (O/U) statistic is a gross indicator of the tendency to show higher or lower average confidence than can be justified by average accuracy. It varies from – 1 to + 1, with negative O/U scores reflecting underconfidence and positive scores showing overconfidence. The normalized resolution index (NRI) shows the ability to discriminate accurate from inaccurate decisions based on participant’s confidence judgments and can range from 0 (lowest resolution) to 1 (perfect discrimination). Table C1 shows the calibration indices for each lineup member position in Experiment 1. Choosers who were presented with both front and right-profile lineup images (same + additional condition) showed higher resolution compared to choosers who only saw lineup members from an angle different from encoding. Non-choosers in the different pose condition were significantly more overconfident compared to non-choosers who saw lineup member in the same pose as at encoding. The 95% CIs for the other calibration indices overlap.

| Table C1  *Calibration measures for each lineup member position split by choosers and nonchoosers in Experiment 1* | | | | | | |
| --- | --- | --- | --- | --- | --- | --- |
|  | Same pose | | Different pose | | Same + additional pose | |
|  |  | 95% CI |  | 95% CI |  | 95% CI |
| Choosers |  | |  | |  |  |
| *C* | .053 | .035; .071 | .062 | .041; .083 | .036 | .022; .051 |
| *O/U* | .216 | .179; .254 | .237 | .196; .278 | .186 | .149; .223 |
| *NRI* | .162 | .103; .220 | .069 | .027; .110 | .171 | .112; .231 |
| Nonchoosers |  | | | |  |  |
| *C* | .018 | .006; .029 | .037 | .019; .054 | .013 | .002; .024 |
| *O/U* | .016 | -.027; .059 | .107 | .061; .154 | .039 | -.004; .082 |
| *NRI* | .023 | -.007; .053 | .017 | -.007; .041 | .045 | .005; .085 |

***Experiment 2***

Figure C2 presents the confidence-accuracy relationships for the high and low pose-reinstatement groups in Experiment 2. CAC analyses were performed in the same manner as Experiment 1. Suspect IDs made with high confidence were higher in accuracy than those made with medium confidence, which in turn were higher in accuracy than those made with low confidence. This is true for those in the high pose-reinstatement group and low pose-reinstatement group. There was a reliable difference between pose reinstatement groups, with high pose reinstatement demonstrating relatively low confidence given their level of accuracy.

Considering the whole confidence scale, there is a larger difference between the conditions in Experiment 2 compared to Experiment 1. In Experiment 1 proportion correct in the different pose condition is not reliably different from the other two conditions, and there is no consistent ordering of the conditions across the confidence levels. One possible explanation is that, in Experiment 1, participants who saw a different pose than they studied may might have found the task was relatively difficult, and so generally adjusted their confidence judgements accordingly to account for their lower accuracy (d’). This would have allowed them to achieve a similar proportion correct as those in the same pose and same + additional pose conditions. Indeed, by looking at the ROCs in Figure 2, participants responded more conservatively for higher confidence IDs in the "different" condition compared the same and same + additional conditions, because the leftmost ROC points are closer to (0,0) in ROC space in the different condition. On the other hand, those in the Experiment 2 had the choice about what they wanted to do at test. Those who chose not to rotate much (i.e., those in the low pose reinstatement group) might not have been aware that they would perform better if they reinstated pose to an even greater degree. Therefore, those in the low pose reinstatement group might not adjust their confidence judgements to account for their lower accuracy (d’) compared to those in the high pose reinstatement group, and so they achieved a lower proportion correct at each level of confidence compared to the high pose reinstatement group. A different possibility is that in Experiment 2 rotating the faces more, and sampling more features, led to more uncertainty in the high compared to low pose reinstatement group.


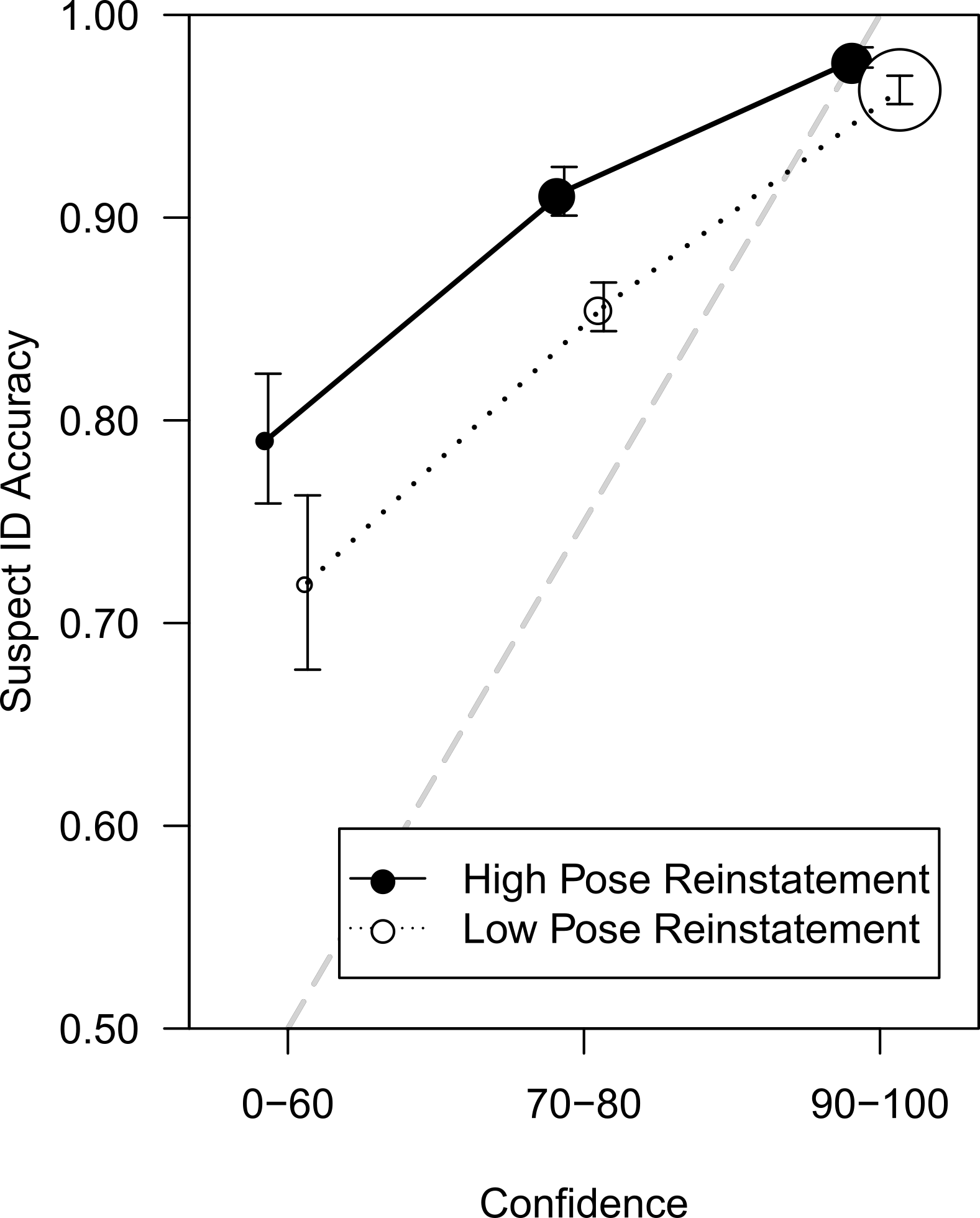


*Figure C2*. Confidence accuracy characteristic curves (CACs) for the high pose-reinstatement and low-pose reinstatement participants. The size of the symbols represents the number of suspect IDs at a given level of confidence relative to the total number of suspect IDs collapsed across all levels of confidence. Error bars reflect +1 SE.

Table C2 presents calibration indices for high and low pose reinstatement participants in Experiment 2. Low pose reinstatement choosers showed significantly worse calibration and higher overconfidence compared to choosers who reinstated the pose of the lineup face to a high degree. The confidence intervals for the other indices overlap. Note the calibration statistics below include filler identification data, whereas the CACs do not.

| Table C2  *Calibration measures for the high pose reinstatement and low pose reinstatement participants split by choosers and nonchoosers in Experiment 2* | | | | |
| --- | --- | --- | --- | --- |
|  | High pose reinstatement | | Low pose reinstatement | |
|  |  | 95% CI |  | 95% CI |
| Choosers |  | |  | |
| *C* | .025 | .011; .039 | .070 | .047; .093 |
| *O/U* | .148 | .107; .189 | .251 | .207; .294 |
| *NRI* | .158 | .098; .219 | .153 | .087; .219 |
| Nonchoosers |  | | | |
| *C* | .015 | .000; .030 | .012 | .001; .022 |
| *O/U* | -.023 | -.073; .027 | .015 | -.037; .067 |
| *NRI* | .032 | -.007; .071 | .032 | -.017; .081 |

References

1. Robin, X., Turck, N., Hainard, A., Tiberti, N., Lisacek, F., Sanchez, J. C., & Müller, M. pROC: An open-source package for R and S to analyze and compare ROC curves. *BMC Bioinformatics. 12*, 77. http:// dx.doi.org/10.1186/1471-2105-12-77 (2011).
2. Cumming, G. Inference by eye: reading the overlap of independent confidence intervals. Stat Med. 28, 205-20. doi: 10.1002/sim.3471 (2009).
3. Wixted J.T., Vul E., Mickes L, Wilson, B. M. Models of lineup memory. Cogn Psychol. 105, 81-114. doi: 10.1016/j.cogpsych.2018.06.001 (2018).
4. Seale-Carlisle, T. M., Wetmore, S. A., Flowe, H. D., & Mickes, L. Designing police lineups to maximize memory performance. ***J****. Exp. Psychol. Appl.* **25,** 410–430. 10.1037/xap0000222 (2019).
